# E-therapists’ views on the acceptability and feasibility of an internet-administered, guided, low-intensity cognitive behavioural therapy intervention for parents of children treated for cancer: A qualitative study

**DOI:** 10.1177/20552076241260513

**Published:** 2024-06-05

**Authors:** Christina Reuther, Johan Lundgren, Maria Gottvall, Johan Ljungberg, Joanne Woodford, Louise von Essen

**Affiliations:** 1 Healthcare Sciences and e-Health, Department of Women's and Children's Health, 8097 Uppsala University, Uppsala, Sweden; 2 Division of Nursing Sciences and Reproductive Health, Department of Health, Medicine and Caring Sciences, 4566 Linköping University, Norrköping, Sweden; 3 Department of Health Sciences, The Swedish Red Cross University College, Huddinge, Sweden

**Keywords:** Cognitive behavioural therapy, e-therapist, internet-administered intervention, digital psychological intervention, cancer, qualitative research

## Abstract

**Background:**

Childhood cancer treatment completion can be a period of vulnerability for parents and is associated with mental health difficulties such as depression and anxiety. We developed an internet-administered, guided, low-intensity cognitive behavioural therapy-based self-help intervention (EJDeR) for parents delivered on the U-CARE-portal (Portal). The acceptability and feasibility of EJDeR and study procedures were examined using a single-arm feasibility trial (ENGAGE). Results indicated that EJDeR and ENGAGE study procedures are acceptable and feasible, however, a need for clinical and technical modifications to EJDeR and refinements to ENGAGE study procedures was identified.

**Objectives:**

This study aimed to explore the acceptability and feasibility of EJDeR and ENGAGE study procedures from the perspective of e-therapists to inform clinical and technical modifications to EJDeR and refinements to study procedures prior to progression to a superiority randomised controlled trial.

**Methods:**

We conducted semi-structured interviews with 10 e-therapists. Data were analysed using manifest content analysis.

**Results:**

We identified three categories relating to the acceptability and feasibility of EJDeR: (a) *Support to e-therapists* (subcategories: Clinical supervision and Technical difficulties); (b) *Guidance to parents* (subcategories: Support protocols and Synchronous communication); and (c) *Content* (subcategories: Relevancy of the intervention and Pacing of the intervention). We identified four categories relating to the acceptability and feasibility of study procedures: (a) *Recruitment and training of e-therapists* (subcategories: Definition of the role and Training program); (b) *Retention of parents* (subcategories: Parent suitability and screening and Frequency of weekly Portal assessments); (c) *Retention of e-therapists* (subcategories: Administrative requirements and Communication with the research team); and (d) *The Portal*.

**Conclusions:**

EJDeR and study procedures were considered acceptable and feasible, however, clinical and technical modifications and refinements to study procedures were suggested to enhance acceptability and feasibility. Results may also inform implementation considerations for both EJDeR and other similar digital psychological interventions.

**Trial registration number:**

ISRCTN 57233429

## Introduction

Childhood cancer is a leading cause of disease burden among children.^1^ Worldwide around 400,000 children and adolescents up to age 19 are diagnosed with cancer yearly^2^ of which approximately 370 are diagnosed in Sweden.^3^ Given significant medical advances, more than 80% of children in high-income countries treated for cancer are cured.^4^ Nevertheless, cancer treatment completion can be a period of psychological vulnerability for parents,^5–7^ with a subgroup reporting psychological^8–10^ and socioeconomic difficulties after the end of treatment.^11,12^ Psychological and socioeconomic difficulties include productivity losses,^13^ fear of relapse,^14,15^ depression,^16^ anxiety,^17^ sleep disturbances, and posttraumatic stress symptoms (PTSS).^6^ Despite these difficulties, there is a lack of support for parents after the end of treatment^7^ and parents report an unmet need for psychological support.^17,18^ As well as a lack of psychological support, parents may experience additional barriers to accessing support including, guilt, prioritising the needs of the child, and lack of time.^19,20^

### A possible solution

Low-intensity cognitive behavioural therapy (LICBT) represents a possible solution to improve access to psychological support which is being implemented by healthcare services globally.^21,22^ LICBT interventions are delivered via self-help materials, for example in print or digital format, including internet-administered CBT (iCBT),^23^ and have been shown to reduce symptoms across a range of mental health difficulties, including depression and anxiety.^24,25^ Interventions guided by trained professionals are associated with larger effect sizes for both depression^26^ and anxiety^27^ than unguided iCBT. Further, guided iCBT versus traditional face-to-face psychological interventions show equivalent overall effects in adult populations.^28,29^

Given the potential of an internet-administered, guided, LICBT-based self-help intervention for parents of children who have completed cancer treatment, following Phase I (development) of the Medical Research Council (MRC) complex intervention framework,^30^ and working alongside Parent Research Partners, we developed the EJDeR intervention (EJDeR [int**E**rnetbaserad s**J**älvhjälp för föräl**D**rar till barn som avslutat en behandling mot canc**eR**]).^31–35^ EJDeR is tailored towards depression and generalised anxiety disorder (GAD), common psychological difficulties experienced by parents of children treated for cancer,^10,14^ and includes four LICBT modules: introduction and psychoeducation (IPE), behavioural activation (BA), worry management (WM), and relapse prevention (RP). Parents are guided to use the intervention by an e-therapist over 12 weeks. EJDeR was delivered on the U-CARE-portal (Portal), a web-based platform, designed to deliver digital healthcare interventions and support digital study procedures (e.g. online consent and data collection).^23^

### The ENGAGE feasibility trial

Following Phase II (feasibility) of the MRC complex interventions framework^30,36^ we conducted the single-arm feasibility trial ENGAGE.^37^ The aims of ENGAGE were to examine methodological (e.g. recruitment and retention), procedural (e.g. data collection instruments and procedures), and clinical (e.g. intervention acceptability and feasibility) uncertainties to prepare for the design and conduct of a superiority randomised controlled trial (RCT) of EJDeR plus usual care (UC) versus UC (the CHANGE trial). Findings indicated that methods, EJDeR, and ENGAGE study procedures are acceptable and feasible, and progression to the CHANGE trial is warranted.^38^ However, the findings also suggested the need for some clinical and technical modifications to EJDeR and refinements to ENGAGE study procedures prior to progression. Regarding EJDeR: (a) the overall adherence rate was 47.9% slightly lower than the progression criteria (≥50%); (b) adherence rates differed by the first LICBT module opened and by gender, with 77% adhering to BA and 50% adhering to WM, with adherence to WM dropping to 42% for fathers; and (c) psychology program student e-therapists did not have time to provide guidance due to parent caseloads being higher than anticipated.^38^ Regarding ENGAGE study procedures: (a) Portal outcome assessment completion was marginally under the progression criteria of ≥70% for post-treatment (12 weeks) as well as follow-up (6 months) and (b) completion rates for weekly Portal assessments during the intervention were low (decreasing over time from 65.7% to 38.9%). Given the intervention adherence rate was slightly lower than the progression criteria, student e-therapists lacked adequate time to support parent caseloads, and challenges were experienced with Portal outcome assessment completion, some clinical and technical modifications of EJDeR and refinements to study procedures are warranted.

### E-therapist involvement

Guidelines concerning the development of complex healthcare interventions recommend stakeholder involvement, including healthcare professionals involved in intervention delivery.^39^ Intervention refinements and improvements are recommended to be made throughout the feasibility/pilot testing and evaluation phases^39^ and collaboration with healthcare professionals supporting digital psychological interventions is recommended to improve engagement.^40^ Therefore, to further inform clinical and technical modifications to EJDeR and refinements to ENGAGE study procedures it is essential to obtain feedback from e-therapists guiding the intervention. Given that e-therapists play a crucial role in the delivery of guided digital psychological interventions, understanding their perspectives on the acceptability and feasibility of digital psychological interventions (e.g. attitudes towards technology, perceptions of therapeutic efficacy, and experiences in guiding psychological interventions via digital platforms) is essential to inform intervention modifications. Importantly, existing literature exploring e-therapists’ experiences of digital psychological interventions suggests that such interventions are acceptable for both e-therapists and patients.^41–45^ Benefits of digital psychological interventions from the perspectives of e-therapists include increased accessibility and flexibility, allowing CBT to be delivered to individuals who may face barriers to attending traditional face-to-face therapy, such as geographical constraints and stigma associated with seeking mental health support.^46,47^ Additionally, digital psychological interventions offer opportunities for personalised and interactive experiences, enabling e-therapists to tailor interventions to meet the unique needs of each participant.^48^

Despite perceived benefits, several challenges have also been highlighted^44,45^ including therapist's lack of knowledge and need for training and supervision^44,49,50^; difficulties tailoring interventions to patient needs^45^; time pressure^45,49,51^; and technical difficulties.^45,52^ Understanding potential challenges experienced by e-therapists may help enhance the acceptability and feasibility of EJDeR and refine study procedures to be used in the CHANGE trial. Further, despite the evidence base, research suggests that iCBT is implemented in only a few primary care organisations in Sweden^53^ with a number of implementation barriers identified.^54^ Understanding factors associated with the acceptability and feasibility of EJDeR, such as the usability of the Portal and the adequacy of training and support, from the perspective of e-therapists (i.e. healthcare professionals) may facilitate our understanding of potential barriers and facilitators related to future implementation^55^ should EJDeR be found to be clinically cost-effective. For example, involving professional stakeholders may help us to understand potential barriers and facilitators to intervention use for both e-therapists and parents. The findings may therefore also be used to optimise the future implementation potential of EJDeR, and other similar interventions, in real-world settings. Thus, this study aims to explore the acceptability and feasibility of EJDeR and ENGAGE study procedures from the perspective of e-therapists to inform clinical and technical modifications to EJDeR and refinements to study procedures prior to progression to the CHANGE trial.

## Methods

### Study design

We conducted an embedded qualitative study using semi-structured interviews to explore the acceptability and feasibility of EJDeR and ENGAGE study procedures from the perspective of e-therapists. This study is reported in accordance with the consolidated criteria for reporting qualitative research (COREQ) was adopted (Appendix 1).^56^

### Research team and reflexivity

Interviews were conducted by a female research assistant with an MSc in economics who was not involved in the recruitment, training, or coordination of e-therapists, and had some experience and training in conducting qualitative interviews. Data analysis was conducted by JL, a male researcher with a PhD in Medical Sciences; JLL, a male research assistant with an MSc in Public Health; and CR, a female PhD student with an MSc in Public Health. CR worked as a project coordinator in ENGAGE and thus had some pre-existing relationships with the e-therapists. Peer examination was provided by LvE, a female professor in Healthcare Sciences and principal investigator for ENGAGE; MG, a female senior lecturer in Public Health and PhD in Medical Sciences; and JW, a female assistant professor in Healthcare Sciences and PhD in Psychology, who are all experienced in conducting, teaching, and supervising qualitative research. JW also provided training in the LICBT intervention to e-therapists.

### Ethical approval

ENGAGE was originally approved by the Regional Ethical Review Board in Uppsala, Sweden (Dnr: 2017/527), with an amendment obtained for this study from the Swedish Ethical Review Authority (Dnr: 2020-05479). The research was conducted following the Helsinki Declaration, and verbal informed consent was obtained prior to interviews.

### Participant selection

Ten e-therapists were recruited to guide parents using EJDeR between April and May 2020 with an additional three e-therapists recruited in September and November 2020. E-therapists were recruited via posters at the Department of Psychology (Uppsala University), advertisements on Swedish clinical psychology Facebook groups, and word-of-mouth. E-therapists were psychology programme students, in at least their fourth year of study including a term of advanced studies in CBT, who had not yet gained an accredited mental health professional qualification (*n* = 10), accredited clinical psychologists (*n* = 2), and a CBT therapist with an MSc in Psychology (*n* = 1). Twelve e-therapists were invited to be interviewed via e-mail (1 e-therapist was not invited due to researcher error), 2 e-therapists declined to be interviewed, and the remaining 10 e-therapists participated in the semi-structured interviews.

### Setting

ENGAGE was a single-arm feasibility trial, with data collected at baseline, post-treatment (12 weeks), and follow-up (6 months) with an embedded mixed-methods process evaluation, and coordinated from Uppsala University, Sweden.^37,38^ In addition, weekly Portal assessments of self-reported depression (Patient Health Questionnaire [PHQ-9]),^57^ PTSS (Post-traumatic Stress Disorder Checklist for DSM-5 [PCL-5],^58^ and the DSM-IV [PCL-C]),^59^ experiential avoidance (Acceptance and Action Questionnaire, 6-items [AAQ-6]),^60^ and depressed inactivity (Behavioural Activation for Depression Scale [BADS])^61^ were collected during the 12-week intervention period. Eligible parents were: (a) a parent of a child diagnosed with childhood cancer (0–18 years) who completed treatment 3 months to 5 years previously; (b) a resident in Sweden; (c) able to read and understand Swedish; (d) able to access e-mail, internet and Bank-ID (a Swedish citizen authentication system); and (e) self-reporting a need for psychological support related to the child's cancer. E-therapists guided EJDeR onsite at Uppsala University. EJDeR was developed for parents of children treated for cancer 3 months to 5 years previously and provided over 12 weeks. A detailed description of EJDeR following the Template for Intervention Description and Replication (TIDieR)^62^ checklist has been published,^23,63^ and a summary of main intervention features is provided below.

#### Intervention content

**Figure 1.**
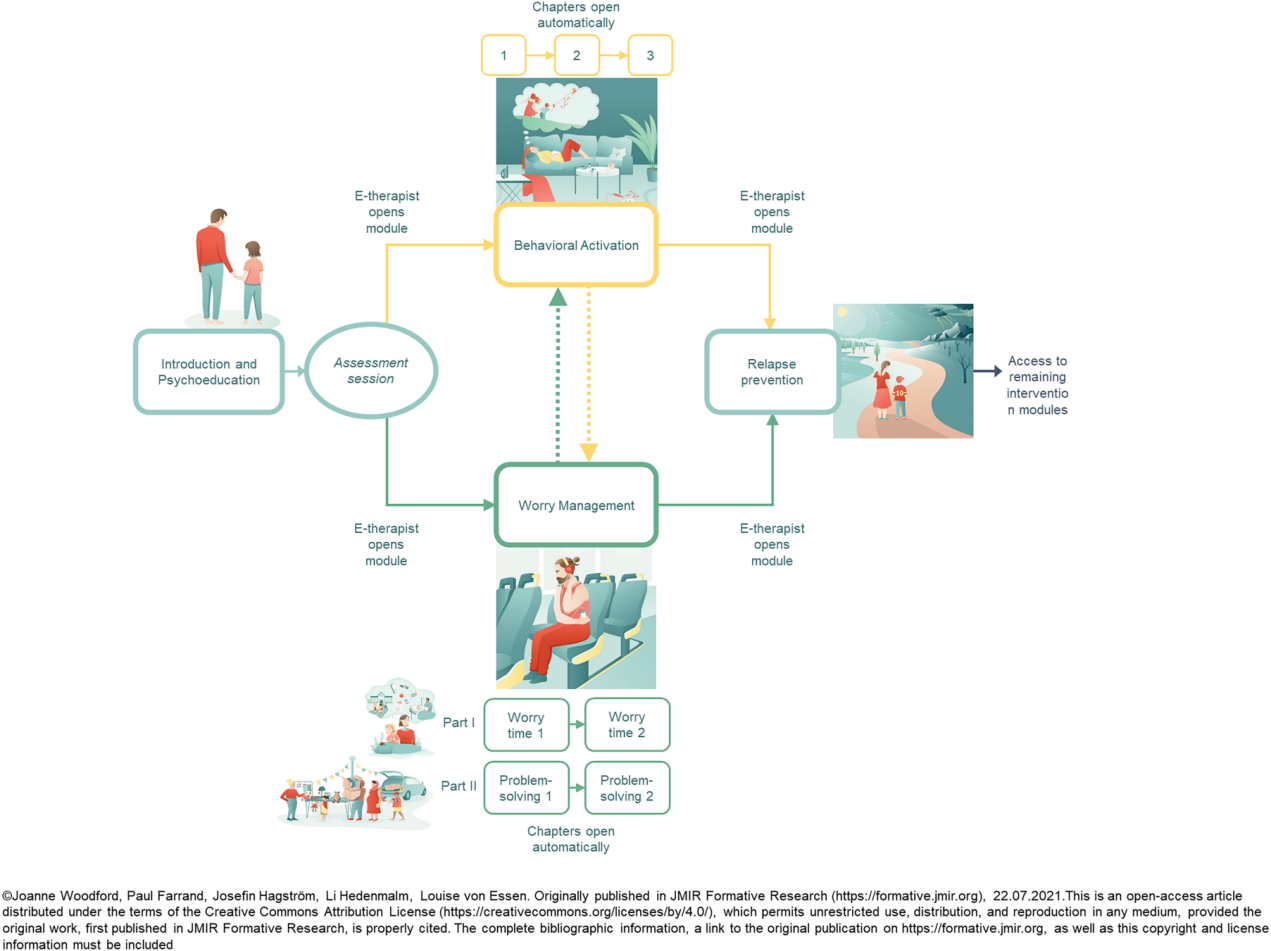
An overview of the structure of EJDeR.

EJDeR is a multimedia digital intervention, delivered on the Portal, and includes text, illustrations, films, and audio files, and can be accessed on computers, smartphones, and tablets. In-module online exercises and weekly homework online exercises (available to print via PDF) were included to facilitate intervention engagement. EJDeR consisted of four modules: (a) IPE (parents were provided with information about psychological distress in the context of being a parent of a child treated for cancer, including case vignettes and goal-setting exercises), (b) BA (parents were supported to gradually engage with pleasurable, necessary, and routine activities they have stopped doing), (c) WM (parents were supported to use problem solving to address practical worries that are important and can be solved and worry time for hypothetical worries, that is worries related to past events, things that might happen in the future, or things that cannot be controlled) and (d) RP (parents were supported to identify warning signs of relapse and develop a staying-well toolkit). BA is a LICBT technique for depression,^64^ and WM is a LICBT technique for GAD.^65,66^ Parents initially worked with IPE and after an initial assessment session with an e-therapist, parents were provided with access to either BA or WM, dependent on their main difficulty (i.e. depression or GAD). Upon completion of BA or WM, a collaborative decision was made between the e-therapist and the parent on whether to use the remaining LICBT technique. All parents were provided with access to the RP module at the end of EJDeR. An overview of the structure of EJDeR is shown in Figure 1.

#### E-therapist guidance

EJDeR was guided by e-therapists via telephone, video-conferencing, and written messages via the Portal. An initial assessment session (≈45 minutes), informed by existing structured support protocols,^63^ was held via telephone or video-conferencing (dependent on parent preference). During the initial assessment session, a collaborative decision was made between the e-therapist and the parent to work with either BA or WM. After the initial assessment, e-therapists gave parents access to the relevant module. Parents were subsequently provided with weekly guidance via written messages via the Portal (≈20–30 minutes per parent each week), informed by an existing brief check-in support protocol^63^ and the internet CBT Therapist Rating Scale.^67,68^ E-therapists provided at-need written messages via the Portal if requested. A mid-intervention booster session (≈30–45 minutes) via telephone or video-conferencing, following a support protocol, was provided to parents to review and assess progress, provide encouragement and motivation, and problem-solve any difficulties experienced.^23^ Structured support protocols for the initial assessment session, written messages via the Portal, and mid-intervention booster sessions were provided to e-therapists in English. Paper-based clinical records were used to record the date of guidance/contact attempt, summarise treatment provided and treatment progress, continued treatment plan, and details of signposting to other care if applicable. Paper-based clinical records were stored in a secure locked cabinet at Uppsala University. Portal-generated automatic notifications were originally sent to e-therapists when parents sent written messages via the Portal, submitted in-module online exercises and weekly homework online exercises, and before scheduled initial assessment and mid-intervention booster sessions. However, due to some e-therapists reporting receipt of notifications as stressful and time-consuming, this Portal feature was turned off shortly after commencing ENGAGE.

#### E-therapist training program

A 2-day training program was provided in English by JW and a professor external to the research team, both LICBT experts from the United Kingdom (UK), a Swedish licensed psychologist, and two research assistants (MSc Psychology). Training focused on: (a) what is LICBT; (b) BA and WM treatment protocols; (c) the structure of EJDeR; (d) the initial assessment session, mid-intervention booster session, and brief check-in support protocols; and (e) the Portal. E-therapists had access to a Portal handbook via Google Docs. After the training program, e-therapists were provided with three written ‘patient scenarios’, depicting parents who had sent a written message via the Portal to their e-therapist concerning difficulties working with BA and the strategies used in WM (worry time and problem-solving). E-therapists were required to write a message in response to each ‘patient scenario’ and received feedback from JW, the LICBT expert from the UK, and the Swedish licensed psychologist. E-therapists had access to videos of the training sessions, PowerPoint slides used to accompany the training sessions, support protocols, and relevant scientific literature via Padlet (a free online collaboration tool: https://padlet.com/).

#### E-therapist supervision

Weekly group supervision (≈2 hours) was provided by a Swedish licensed psychologist with expertise in iCBT. The supervision primarily focused on clinical skills and included case discussions and skill development (i.e. developing and maintaining competence in BA and WM treatment protocols and using the telephone, video-conferencing, and written messages via the Portal to support LICBT working).^23^ Other aspects such as potential risk issues, intervention period extensions, and the need to signpost parents to other types of support were discussed. On-demand individual supervision was provided if required.

### Sample description

To facilitate the interpretation of supporting quotations, selected characteristics for e-therapists with pseudonyms are provided in Table 1. All e-therapists were female (*n* = 10), of which the majority (*n* = 7) were psychology programme students and the remaining e-therapists were licensed clinical psychologists (*n* = 2) and a CBT therapist (MSc Psychology: Focus CBT) (*n* = 1).

**Table 1.** Sample characteristics (*n* = 10).

Name	Gender	Mental health professional qualification*	Number of parents guided	Modules guided†
Isabelle	Female	Qualified	≥5	IPE, BA, WM, RP
Wilma	Female	Qualified	≥5	IPE, BA, WM, RP
Emily	Female	Qualified	≥5	IPE, BA, WM, RP
Oliva	Female	Not yet qualified	≥5	IPE, BA, WM, RP
Anna	Female	Not yet qualified	<5	IPE, BA, WM, RP
Astrid	Female	Not yet qualified	<5	IPE, WM, RP
Frida	Female	Not yet qualified	<5	IPE, BA, WM
Alma	Female	Not yet qualified	<5	IPE, BA, WM, RP
Madeleine	Female	Not yet qualified	<5	IPE, BA, WM
Sophie	Female	Not yet qualified	<5	IPE, WM, RP

*Note.* Pseudonyms are used to protect parents’ confidentiality; *Qualified = Licensed clinical psychologists or CBT therapists; Not yet qualified = Psychology program students; †IPE = Introduction & psychoeducation; BA = Behavioural activation; WM = Worry management; RP = Relapse prevention.

### Data collection

Interviews were conducted in Swedish following a semi-structured interview guide (see Appendix 2) exploring e-therapists’ perceptions regarding (a) the content of EJDeR (i.e. LICBT techniques, exercises, barriers and facilitators to use, and relevancy to parents); (b) the Portal (i.e. usability); (c) providing guidance (i.e. initial assessment session, weekly guidance via written messages via the Portal, and mid-intervention booster session); (d) training and supervision; and (e) study procedures (i.e. communication with the research team and technical support). Interviews were conducted between January and March 2021. After providing verbal informed consent e-therapists were interviewed over the telephone (*n* = 8) or face-to-face at Uppsala University (*n* = 2), based on preference. Interviews lasted a mean of 56 minutes (SD 0.01, range 36–81) and no repeat interviews were conducted. Interviews were audio recorded on an Olympus Digital Voice Recorder WS-853.

### Data processing and analysis

Interviews were transcribed verbatim in Swedish by a professional transcriber, with identifying information (e.g. names) removed. Microsoft Word and Excel were used to support data analysis, and all data analysis activities were recorded in an audit trail (Microsoft Excel). Manifest content analysis with an inductive approach^69^ was adopted, with an example of the analysis process provided in Table 2. Authors JL, JLL, and CR participated in an analysis training workshop with JW. JL, JLL, and CR independently read the transcripts and identified, condensed and coded meaning units, remaining close to the manifest content. Next, JL, JLL, and CR individually performed a preliminary categorisation of codes into categories and subcategories with a low degree of interpretation. Subsequently, a data analysis meeting was held with authors LvE, MG, JL, JLL, and CR to establish the credibility and dependability of the analysis. During the meeting, JL, JLL, and CR presented identified categories and subcategories on Post-it notes with categories and subcategories covering similar content groups. A decision was made to present the analysis in two overarching themes relating to study aims. After revisions were made, categories and subcategories were reapplied to all interviews by JL, JLL, and CR independently to ensure a credible foundation in the data. Category and subcategory descriptions were written by CR in Swedish and sent to LvE and MG for peer examination. After further revisions, descriptions were translated from Swedish to English and presented to MG and JW for further peer examination. Supporting quotations were selected by JL, JLL, and CR, translated from Swedish to English by a native Swedish speaker (MG) and reviewed by a native English speaker (JW).

**Table 2.** Example of the analysis process.

Meaning unit	Condensed meaning unit	Code	Subcategory	Category	Theme
I think the length [of EJDeR] is just right actually. I can’t really see any point in dragging it out any further. Then there might be the risk that you [parents] might put off doing the tasks or logging in, so it's probably good that it's compacted into 12 weeks …	The length is just right. If you drag it out, there is a risk parents will delay doing the tasks or logging in.	The length of EJDeR is good	Pacing of the intervention	Content	Acceptability and feasibility of EJDeR
I found it was a bit of a hassle to add new homework exercises and adding new worksheets for behavioural activation [onto the Portal]. I found it a bit complicated and it wasn’t obvious how to do it.	It was a hassle to add homework exercises and worksheets.	The Portal is not user-friendly	–	The Portal	Acceptability and feasibility of ENGAGE study procedures

#### Trustworthiness

Strategies to establish trustworthiness^70,71^ included the use of an audit trail (i.e. the data analysis process and analysis decisions were documented in a Microsoft Excel spreadsheet), a detailed description of the analysis process, peer examination (i.e. research procedures and findings discussed with research team members not involved in analysis), and researcher triangulation (i.e. involving several researchers in coding, analysis and interpretation). To strengthen the credibility of the findings, supporting quotations are provided. A description of e-therapist characteristics, EJDeR and the ENGAGE study setting are presented to provide readers with an understanding of the context and facilitate the transferability of the findings.

## Results

Table 3 provides an overview of themes, categories, and subcategories. The analysis resulted in two overarching themes relating to study aims: (a) Acceptability and feasibility of EJDeR and (b) Acceptability and feasibility of ENGAGE study procedures.

**Table 3.** Overview of themes, categories, and subcategories.

Themes	Categories	Subcategories
Acceptability and feasibility of EJDeR	Support to e-therapists	Clinical supervision
		Technical difficulties
	Guidance to parents	Support protocols
		Synchronous communication
	Content	Relevancy of the intervention
		Pacing of the intervention
Acceptability and feasibility of ENGAGE study procedures	Recruitment and training of e-therapists	Definition of the role
		Training program
	Retention of parents	Parent suitability and screening
		Frequency of weekly Portal assessments
	Retention of e-therapists	Administrative requirements
		Communication with the research team
	The Portal	-

### Acceptability and feasibility of EJDeR

The first overarching theme relates to e-therapists’ perceptions of the acceptability and feasibility of EJDeR and includes three categories: *Support to e-therapists, Guidance to parents,* and *Content*.

#### Support to e-therapists

The category includes two subcategories: Clinical supervision and Technical difficulties.

**Clinical supervision.** Overall, e-therapists were satisfied with the clinical supervision provided, finding weekly supervision to be supportive, and e-therapists appreciated quick responses to ‘on-demand’ enquiries:

‘*It* [clinical supervision] *has been really great … we’ve also had the opportunity to contact the supervisor over the Portal to get quick answers about minor things, so it's really felt like enough*’. (Frida)

However, the perceived need for clinical supervision frequency varied by nature of e-therapists’ previous experience, caseload size, and how active parents were in the intervention. For example, more experienced e-therapists or those with small caseloads and/or inactive parents considered weekly supervision too frequent.

**Technical difficulties**. Support from the research team for technical difficulties was perceived as accessible and responsive. The Portal handbook was considered helpful, with answers to most technical enquires related to guiding EJDeR found in the text:

‘*It* [the Portal handbook] *was very good. It was easy to find everything and you really got help from there*’. (Anna)

However, improvements were suggested such as reducing the amount of text and combining technical information in the Portal handbook with clinical information provided via Padlet to facilitate navigation and minimise confusion.

E-therapists expressed a need for ‘technical supervision’ in using the Portal. A research team member attending clinical supervision was suggested as being beneficial, given the clinical supervisor was not always able to answer technical questions and contacting the research team was perceived as time-consuming.

#### Guidance to parents

The category includes two subcategories: Support protocols and Synchronous communication.

**Support protocols.** Support protocols for the initial assessment session, weekly guidance via written messages via the Portal, and the mid-intervention booster sessions were considered helpful, providing e-therapists with enough information to guide parents to use EJDeR. However, some found the protocols overly mechanical and structured, hindering communication and the development of a therapeutic relationship with parents. E-therapists expressed a desire to adapt protocols to further personalise the guidance provided:

‘*I noticed over time, when you had established contact with parents, that it felt a bit robotic to begin* [each written message] *with “hello, this is your parent guide, I’m writing to give you the weekly feedback”. It was a bit square, perhaps. I would have liked a bit more space to make the messages a bit more personal*’. (Alma)

Difficulties were voiced concerning adherence to protocols when parents were not using EJDeR as planned, with e-therapists wanting more guidance on how to better engage and motivate inactive parents. Furthermore, protocols only being provided in English was described as a limitation and e-therapists expressed that translation to Swedish would have facilitated communication with the parents.

**Synchronous communication.** The initial assessment and mid-intervention booster sessions, facilitating synchronous communication with parents, were considered valuable, helpful, and were appreciated. E-therapists expressed talking over the telephone and video-conferencing as being easier than providing written messages via the Portal. Video-conferencing was perceived as particularly helpful in building a therapeutic relationship with parents:

‘*… I think you should emphasise having a video call, because it felt like you had much better contact and you could see that there is a real person there. It is not just a voice* [over the telephone]* … I felt that we had a better relationship* [with parents supported over video-conferencing] *than with those I only spoke with on the phone*’. (Olivia)

E-therapists mentioned that additional follow-up or booster sessions were needed and wanted by both e-therapists and parents. They also considered efforts to reach out to parents via SMS or telephone to motivate them to use EJDeR as being essential for engagement and retention. These efforts were perceived as more effective than sending written messages via the Portal. Efforts to reach out to parents via SMS or telephone were also considered to facilitate reducing the shame some parents experienced when unable to engage with EJDeR as planned:

‘*I think the contact with e-therapists and being reminded to do things* motivates [the parents] *quite a lot. There can be strong feelings of shame if, for example, parents fell behind on an exercise or had a lot going on and hadn’t prioritised it* [EJDeR]*. But the e-therapist can remove some of the shame by validation*’. (Frida)

Sending an SMS or calling parents who were having difficulties getting started with the intervention was viewed as more effective for engaging parents than sending written messages via the Portal.

#### Content

The category includes two subcategories: Relevancy of the intervention and Pacing of the intervention.

**Relevancy of the intervention.** Overall, e-therapists were satisfied with the content of EJDeR, perceiving it to be informative and relevant. E-therapists described parents as being able to implement the tools and strategies (i.e. LICBT techniques) learned during EJDeR into their daily lives. They however voiced challenges supporting some parents, for example, those who were experiencing difficulties not targeted by EJDeR, for example trauma, grief, and perfectionism:

‘*I think that many* [parents] *suffer from some form of subclinical PTSD, that they have trauma and grief related to the child's illness and that period. And then my clinical feeling is that many parents are “on the edge” or even suffer from burnout*’. (Isabelle)

E-therapists expressed a need to be able to individualise and personalise EJDeR more, for example, by providing parents with more opportunities to share their stories, talk about their cancer experience, and thus enhance intervention relevancy. Further, the rationale for some LICBT techniques and exercises (e.g. BA, goal setting, and worry time) was perceived as unclear by some e-therapists, resulting in difficulties guiding parents. E-therapists also reported a need for additional information in the intervention to help signpost parents to appropriate additional relevant sources of help and support.

**Pacing of the intervention.** E-therapists generally perceived the pacing and overall duration of EJDeR to be appropriate, however, a wish for greater flexibility was voiced. For example, for some parents, the pace of EJDeR was considered too quick and demanding, especially for those experiencing conflicting priorities (i.e. family life, vacation, etc.), which negatively impacted their commitment and engagement. This led to the intervention period being extended beyond 12 weeks for some parents:

‘*… perhaps the parents could have a bit more time than 12 weeks, because there were many who said that it* [EJDeR] *went way too fast and maybe it could be more individualised, so that not everyone needs to have 12 weeks, so there can be greater flexibility perhaps*’. (Emily)

### Acceptability and feasibility of ENGAGE study procedures

The second overarching theme relates to the acceptability and feasibility of ENGAGE study procedures and includes four categories: *Recruitment and training of e-therapists*, *Retention of parents, Retention of e-therapists*, and *The Portal*.

#### Recruitment and training of e-therapists

The category includes three subcategories: Definition of the role and Training program.

**Definition of the role.** E-therapists’ expectations of the role varied. Some saw their role as therapeutic, providing psychological support to parents, while others took on the role as they wanted to gain experience with iCBT and exposure to a clinical research environment. Overall, e-therapists considered the information provided during recruitment as consistent with the role. However, psychology programme students felt the role was more demanding than anticipated, with some experiencing difficulties handling large parent caseloads:

‘*At the beginning* [of the study] *it felt like* [you could] *take as many* [parents] *as you could and you wanted to …*[but] *the project wanted you to take on more and more and more …*’ (Olivia)

**Training program.** Overall, e-therapists considered the training as important and valuable, providing insight into the study, how LICBT is delivered and how to use the Portal. The 2-day duration of the training program was considered reasonable and the possibility to ask questions face-to-face was helpful. The training material was perceived as valuable and clear, and the availability of videos of the training sessions on Padlet was appreciated. However, e-therapists considered it a disadvantage that the training was delivered in English. Misunderstandings concerning EJDeR and support protocols might have been reduced if training and associated materials had been provided in Swedish.

Technical training on how to use EJDeR on the Portal was delivered at the end of the training program and it was considered challenging to retain all information. E-therapists suggested that it could be helpful to access EJDeR on the Portal during the training program thus combining clinical (i.e. LICBT techniques and support protocols) and technical aspects (i.e. using the Portal) of guiding the intervention:

‘*I thought it* [training] *was good and it was just the right length. It was interesting to learn about low-intensity and high-intensity CBT, I think this was new to all of us* [e-therapists]*. Maybe we could have used the Portal ourselves* [during training]*, checked and maybe asked some questions. This would have been such a good thing*’. (Madeleine)

E-therapists also suggested restructuring training topics according to importance. For example, it was suggested that additional training on providing weekly guidance via written messages via the Portal would be helpful, given that providing this type of guidance is very different to delivering face-to-face therapy. Furthermore, e-therapists considered previous work experience to have a positive impact on and facilitate their work.

#### Retention of parents

The category includes two subcategories: Parent suitability and screening and Frequency of weekly Portal assessments.

**Parent suitability and screening.** E-therapists expressed that some parents were not motivated to engage with EJDeR and find time to fit the intervention into their already busy schedules. An e-therapist described how one parent they supported ‘*had a lot to do* [in their private life] *and had felt a lack of motivation to work with the program*’ (Sophie).

It was also mentioned that some parents joined the study for altruistic reasons, rather than for their own psychological need, which negatively impacted their motivation to work with the intervention. Screening at the baseline for depression and anxiety was suggested for future research to avoid including parents without a psychological need and thereby potentially increase retention.

**Frequency of weekly Portal assessments.** E-therapists mentioned that weekly Portal assessments during the intervention were stressful for parents, negatively impacting on intervention engagement as well as study retention. Some parents told their e-therapist that they felt too tired to engage with EJDeR after completing the weekly Portal assessments:

‘*They* [parents] *became a bit confused and stressed by it* [weekly Portal assessments]*. They said that it was often an obstacle to work with the actual EJDeR program. So they completed all these questionnaires, which were very many, and then they were tired and did not have the energy to continue with EJDeR*’. (Emily)

E-therapists suggested that accessing parents’ weekly Portal assessments during the intervention could have facilitated assessment completion by enabling them to provide parents feedback on their weekly assessment scores. Access to parents’ weekly Portal assessment scores could have also informed clinical decision-making.

#### Retention of e-therapists

The category includes two subcategories: Administrative requirements and Communication with the research team.

**Administrative requirements.** E-therapists considered various administrative requirements of the role to negatively impact their own motivation to guide EJDeR and continue to work in the study. For example, the requirement to come to Uppsala University to complete paper-based clinical records was considered time-consuming and unnecessary, with online clinical records suggested as a more convenient solution:

‘*Writing clinical records felt like it took an inordinate amount of time, mostly because one had to go in and write a clinical record for a quarter of an hour once a week. So, there was a lot of time spent just getting there* [Uppsala University] *to do very little work and getting paid very little for it*’. (Astrid)

Further e-therapists expressed that being an hourly employee commuting to the office to complete paper-based clinical records negatively impacted their motivation and commitment to the role, as the commuting time was not paid for.

**Communication with the research team**: Generally, communication with the research team was considered straightforward, with prompt replies conveying availability. Booking rooms was generally considered easy. However, some had encountered difficulties in reaching the research team, despite e-mails and telephone calls:

‘*I don’t remember what it was about, but it was regarding a parent. And I called and called, and e-mailed and e-mailed, and I got no response. And there, I felt like, well, it … it was hopeless*’. (Olivia)

Furthermore, e-therapists found it difficult to articulate challenges in e-mails and would have preferred speaking with research team members either face-to-face or over the telephone. E-therapists mentioned that if they had been more involved in the research team it could have helped them to better understand research team members’ roles and who could support them with specific enquiries, for example, clinical versus practical or technical support.

#### The Portal

Overall, e-therapists were satisfied with the Portal, perceiving it to be user-friendly with the digital format facilitating flexibility to read parents’ work and provide feedback when it suited the e-therapist. However, additional features to facilitate their work were suggested, for example, being able to see a parent's last log-in time and date and the parents’ intervention view. They also considered Portal navigation to be challenging, for example, providing parents with access to modules and adding additional weekly homework online exercises was difficult and time-consuming:

‘[Providing access to modules and adding weekly homework online exercises] *was so unnecessarily complicated … And* [it would be helpful] *if an e-therapist could see the participants’ view, so you can see if it has been done right or not*’. (Wilma)

Weekly online homework exercises needed to be submitted by parents on the Portal for e-therapists to be able to view them. E-therapists described frustration that some parents reported partially completing these exercises but failed to submit them, making it difficult to provide feedback and guidance. They also mentioned challenges with parents receiving written messages via the Portal and feedback on exercises in different parts of the Portal, which was confusing to parents, causing them to miss written guidance from e-therapists. E-therapists also expressed that receiving automated notifications when parents sent written messages via the Portal or submitted exercises, and reminders before initial assessment and mid-intervention booster sessions would have been helpful. Some technical difficulties with seeing and/or hearing parents were experienced with video-conferencing, which was considered frustrating and could lead to shorter initial assessment and mid-intervention booster sessions than required to provide appropriate guidance.

## Discussion

### Main findings

This study aimed to explore the acceptability and feasibility of EJDeR and ENGAGE study procedures from the perspective of e-therapists. Three categories were identified relating to the acceptability and feasibility of EJDeR: *Support to e-therapists, Guidance to parents,* and *Content*. Findings related to *Support to e-therapists* highlighted that clinical supervision and support for technical difficulties were acceptable, however, there was a need for ‘technical’ supervision in using the Portal. Findings related to *Guidance to parents* suggested that structured support protocols were perceived as overly mechanical and difficult to adhere to. Synchronous communication with parents was generally considered more acceptable than written messages via the Portal. Findings related to *Content* suggested the intervention to be informative and relevant, however, some psychological difficulties such as PTSS were not addressed, and a need to better individualise and personalise the intervention was expressed. Four categories were identified relating to the acceptability and feasibility of ENGAGE study procedures: *Recruitment and training of e-therapists, Retention of parents, Retention of e-therapists,* and *The Portal*. The findings suggested that *Recruitment and training of e-therapists* were acceptable, however, needs were identified for providing additional technical training and more focus on written messages via the Portal, especially for parents experiencing difficulties with engagement and motivation. Related to *Retention of parents*, e-therapists perceived low levels of motivation, lack of psychological need, and stress and fatigue associated with weekly Portal assessments during the intervention as negatively impacting parent retention. Findings related to *Retention of e-therapists* indicated that administrative burden associated with completing paper-based clinical records and some difficulties communicating with the research team impacted negatively on their motivation to guide EJDeR and work in the study. Findings related to *The Portal* identified technical difficulties that negatively impacted e-therapists’ work.

#### Comparison with previous literature

E-therapists’ attitudes towards the digital format of EJDeR were largely consistent with the broader digital psychological intervention literature, recognising that the format was convenient, providing flexibility to both e-therapists and parents.^44^ However, in accordance with previous research, e-therapists found the intervention to be too extensive and demanding for some parents^18–20^ and expressed a need for greater flexibility regarding intervention duration and the amount of e-therapist guidance provided to parents to facilitate engagement.^51^

A preference for increased flexibility when following support protocols was identified. Whilst protocols were considered helpful, they were also perceived as overly mechanical and structured, hindering communication and the development of a therapeutic relationship with parents. Other research exploring e-therapists’ perspectives on digital psychological interventions has identified a positive effect of support protocols in preventing therapist drift, sustaining protocol adherence, and increasing the intervention quality.^72^ However, research also suggests providing e-therapists with templates to guide online written support can be viewed as disruptive to the therapeutic relationship, despite e-therapists being able to make template adjustments.^73^ In the present study, support protocols for written messages via the Portal were not designed as templates, but to support e-therapists to (a) review and provide feedback on any submitted exercise, (b) review intervention progress, (c) normalise difficulties, (d) assist with problem solving, (e) set a plan for continued intervention use, and (f) provide encouragement and motivation. The support protocol was informed by the iCBT Therapist Rating Scale^67^ and designed to reduce the use of therapist behaviours identified as undesirable when guiding iCBT.^68^ E-therapists perceiving protocols to be overly mechanical and structured indicates a need for additional training to support protocol adherence, whilst also personalising guidance based on parent needs and intervention progress. The majority of e-therapists supported very few parents and were psychology programme students with minimal experience in delivering traditional face-to-face CBT or guided iCBT. E-therapists with less experience have been found to experience more difficulties with the ﬂexibility and personalisation of digital intervention protocols (i.e. providing online written support), as well as determining patient eligibility, and building a therapeutic relationship in comparison with more experienced therapists.^72^

An additional finding was that e-therapists considered synchronous communication, via telephone and video-conferencing, to be more acceptable and better facilitate building a therapeutic relationship than providing written feedback via the Portal. A need was expressed for providing additional synchronous guidance sessions to parents. Such findings are supported by recent recommendations that sustained engagement with digital health interventions requires some element of human interaction, for example, the provision of ‘virtual’ guidance via telephone or video-conferencing.^74^ However, e-therapists also reported technical challenges with video-conferencing, leading to frustration for both e-therapists and parents. Technical challenges, such as interruptions to video or sound, have been suggested to be problematic in the provision of digital psychological interventions, limiting communication and potentially negatively impacting the therapeutic relationship.^75^

Although e-therapists generally perceived intervention content to be acceptable and relevant, some e-therapists mentioned that some parents experienced psychological difficulties, such as PTSS, that were not being targeted by EJDeR. Our previous research suggests that a subgroup of parents experience clinically relevant PTSS up to five years after treatment,^6,10,76^ and in semi-structured interviews conducted with parents before using EJDeR, concerns were described consistent with PTSS.^77^ Originally, EJDeR was developed to target depression and GAD rather than posttraumatic stress disorder (PTSD) given the lack of evidence base for LICBT for PTSD at the time.^78^ For example, research suggested iCBT had little to no effect on improving PTSD symptoms in comparison with UC in regular healthcare.^79,80^ However, as the evidence base for iCBT for PTSD is growing^80^ future clinical modifications to EJDeR will include psychoeducation and psychological techniques designed to help parents manage PTSS.^81^

To further improve the acceptability of EJDeR for e-therapists, additional Portal features were suggested, for example being able to view parents’ last log-in time and date and parents’ intervention view to better facilitate reviewing parents’ work and progress with EJDeR. A need for enhanced e-therapist intervention management systems, including access to patients’ intervention view, has been identified in other research exploring the acceptability of iCBT from the perspective of e-therapists.^73^ The importance of improving the general usability of the Portal was highlighted, and technical difficulties have been identified as a significant barrier to iCBT uptake,^45,52^ with technology issues, such as functionality, usability, and poor quality of video-conferencing technology negatively impacting acceptability reported elsewhere.^82^ E-therapists also experienced a need for more technical training on how to use the Portal. Other research has identified that e-therapists need time to familiarise themselves with iCBT platforms and receive ongoing clinical and technical supervision^44,49,72^ and more time to practice.^82^

E-therapists perceived weekly Portal assessments during the intervention as stressful for parents, negatively impacting intervention engagement and study retention. Providing e-therapists with access to parents’ weekly Portal assessment scores was suggested as a way to improve retention, by enabling e-therapists to provide parents feedback on their symptoms and inform clinical decision-making. When designing ENGAGE, a decision was made not to collect weekly clinical outcome measures for clinical purposes (i.e. to feedback scores and help inform clinical decision-making) but to inform an embedded process evaluation.^37,38^ However, collecting routine clinical outcomes during interventions, such as self-reported depression and anxiety, has been identified as a core feature of the successful implementation of iCBT in routine healthcare.^83^ Integrating routine clinical outcome collection into psychological interventions is associated with improved patient outcomes^84^ and can be used to personalise intervention delivery, enhance engagement, and reduce drop-out.^85^

### Limitations

First, at the time of the interviews, there was heterogeneity concerning the length of time since e-therapists had worked with EJDeR, with some e-therapists still guiding parents, whereas others had finished their work on the study. Those no longer guiding parents may have found it difficult to remember some aspects of EJDeR and ENGAGE study procedures. Second, the sample size was predetermined by the number of e-therapists working on the ENGAGE study, and it was not possible for data collection or analysis to be guided by principles such as data saturation.^86^ Third, e-therapists responded to advertisements to work on the ENGAGE study which may have impacted on their attitudes towards iCBT more generally, and thus findings may not be transferable to a wider e-therapist workforce who may guide the intervention in the future, should EJDeR be implemented in routine healthcare settings.

Despite these limitations, the study has several strengths, including the use of independent coders, data analysis workshops, peer examination, and keeping an audit trail, strengthening the credibility and confirmability of the findings.

### Implications and future directions

To enhance the acceptability and feasibility of EJDeR and ENGAGE study procedures’ findings will be used to inform clinical and technical modifications to EJDeR and refinements to proposed study procedures for the CHANGE trial, provided in Table 4.

**Table 4.** Clinical and technical modifications made to EJDeR and refinements to ENGAGE study procedures to be used in the CHANGE trial.

Acceptability and feasibility of EJDeR	Clinical and technical modifications made to EJDeR
Support and guidance	Reduction of the Portal handbook text
	Provision of clinical and technical information to e-therapists in the same handbook
	Provision of technical supervision to e-therapists
	Provision of additional training and practice in how to use structured support protocols in a flexible way
	Provision of additional training on how to engage and motivate inactive parents
	Translation of structured support protocols into Swedish
	Provision of an additional follow-up support session via telephone or video-conferencing to parents
Content of EJDeR	Provision of psychoeducation and techniques to manage post-traumatic stress symptoms
	Clarification of the rationale for LICBT techniques in the intervention and e-therapist training materials
	Provision of information and signposting for difficulties parents may experience not targeted by the intervention
Acceptability and feasibility of study procedures	Refinements to ENGAGE study procedures to be used in the CHANGE trial
Recruitment and training	Recruitment of part-time employed therapists to facilitate guiding large parent caseloads, as opposed to psychology programme students
	Delivery of the training program in Swedish
	Provision of additional Portal training
	Provision of additional training on providing weekly guidance via written messages via the Portal
Retention	Development of an enhanced e-therapist intervention management system, i.e. being able to see parents’ last log-in time and date and intervention view and provide access to relevant modules
	Development of an enhanced e-therapist communication system to enable parents to receive all online communication in the same place
	Improvement of video-conferencing system stability and quality

Findings also suggest strategies to increase intervention acceptability and future adoption, should EJDeR be implemented in real-world settings, may need tailoring to e-therapist's prior experience of guiding digital interventions. For example, those with no or minimal prior experience of digital psychological interventions may need additional training and support with technical and usability issues.^82^ A future direction may be to adopt a co-design approach with e-therapists^73^ whereby researchers and e-therapists collaborate to design solutions to identified technical challenges and adaptation of the e-therapist training program.

## Conclusion

The present study aimed to explore the acceptability and feasibility of EJDeR and ENGAGE study procedures from the perspective of e-therapists to inform clinical and technical modifications to EJDeR and refinements to study procedures prior to progression to the CHANGE trial. Overall, EJDeR and ENGAGE study procedures were considered acceptable and feasible, however, several modifications were suggested to inform adaptations to EJDeR and study procedures to be adopted in the CHANGE trial. As well as informing future research, findings may also inform future implementation considerations. For example, clear needs were identified to increase e-therapists’ knowledge of how to support iCBT interventions. Implementation strategies such as interactive training, education outreach visits, provision of ongoing training and supervision, and fostering a collaborative learning environment within implementing organisations^87^ may be required to facilitate future implementation should EJDeR be found to be a clinical and cost-effective solution.

## Supplemental Material

sj-docx-1-dhj-10.1177_20552076241260513 - Supplemental material for E-therapists’ views on the acceptability and feasibility of an internet-administered, guided, low-intensity cognitive behavioural therapy intervention for parents of children treated for cancer: A qualitative study Supplemental material, sj-docx-1-dhj-10.1177_20552076241260513 for E-therapists’ views on the acceptability and feasibility of an internet-administered, guided, low-intensity cognitive behavioural therapy intervention for parents of children treated for cancer: A qualitative study by Christina Reuther, Johan Lundgren, Maria Gottvall, Johan Ljungberg, Joanne Woodford and Louise von Essen in DIGITAL HEALTH

sj-docx-2-dhj-10.1177_20552076241260513 - Supplemental material for E-therapists’ views on the acceptability and feasibility of an internet-administered, guided, low-intensity cognitive behavioural therapy intervention for parents of children treated for cancer: A qualitative study Supplemental material, sj-docx-2-dhj-10.1177_20552076241260513 for E-therapists’ views on the acceptability and feasibility of an internet-administered, guided, low-intensity cognitive behavioural therapy intervention for parents of children treated for cancer: A qualitative study by Christina Reuther, Johan Lundgren, Maria Gottvall, Johan Ljungberg, Joanne Woodford and Louise von Essen in DIGITAL HEALTH
