# Supplementary material for: E-therapists’ views on the acceptability and feasibility of an internet-administered, guided, low-intensity cognitive behavioural therapy intervention for parents of children treated for cancer: A qualitative study
Source: Digit Health. 2024 Jun 5;10:20552076241260513. doi: 10.1177/20552076241260513 (PMC11155313; doi:10.1177/20552076241260513)
Supplement: sj-docx-2-dhj-10.1177_20552076241260513 - Supplemental material for E-therapists’ views on the acceptability and feasibility of an internet-administered, guided, low-intensity cognitive behavioural therapy intervention for parents of children treated for cancer: A qualitative study [file sj-docx-2-dhj-10.1177_20552076241260513.docx]

**Appendix 2.** Semi-structured interview guide for e-therapists

| Topics | Interview questions |
| --- | --- |
| I. Background information | A) How were you recruited to be an e-therapist?  B) What education do you have?  C) What work experience do you have?  D) Did the assignment meet your expectations? If so, why? If not, why not?  E) How many parents have you been an e-therapist to?  Q) How active were the parents? |
| II. Main questions |  |
| EJDeR | A) What do you think of the program?  B) Are any of the CBT techniques particularly good? If yes, which and why?  C) Are any of the CBT techniques less good? If yes, which and why?  D) How did the parents work with the exercises (via the Portal; on paper; or with other aids, such as apps, diary; in their daily lives)?  E) Could the exercises be improved? If so, how?  Q) Did the parents tell you what they thought of the program? If so, what did they tell you?  G) Was there anything that made it difficult for the parents to use the program? If so, what?  H) Was there anything that made it easier for the parents to use the program? If so, what?  I) Do you think the parents worked more with the program than what is seen through submitted modules and exercises?  J) Could the program be improved? If so, how?  K) Do you think the parents had any mental health difficulties that are not covered in the program? If so, which ones?  L) Could the length of the program be improved? If so, how? |
| The Portal | A) How did it work providing feedback via the Portal? Can anything be improved?  B) How did it work adding modules and exercises? Can anything be improved?  C) How did it work to make video calls via the Portal? Can anything be improved?  D) How did it work using the Portal Handbook? Can anything be improved?  E) Were any of the Portal features particularly useful? If yes, which / which and why?  Q) Can any of the Portal features be improved? If yes, which ones and how? |
| E-therapist role | A) How did the initial assessment session work?  B) Was the support protocol for the initial assessment session helpful / sufficient? If not, can something be improved?  C) How did the mid-intervention booster assessment session work?  D) Was the support protocol for the mid-intervention booster assessment session helpful/sufficient? If not, can something be improved?  E) How did the weekly written messages via the Portal work?  Q) Was the support protocol for the weekly written messages via the Portal helpful/sufficient? If not, can something be improved?  G) How did it work to answer questions/messages in the Portal in addition to providing weekly written messages via the Portal?  H) How did you identify which questions/messages were urgent or less urgent to answer?  I) Can the work of the e-therapists be facilitated? If so, how? |
| Training program, EJDeR and the Portal | A) What did you think of the educational material about EJDeR that was provided on Padlet?  B) What did you think of the EJDeR training program that you took part in at the department?  C) What did you think of the education provided about using the Portal that you took part in at the department?  D) Is there any information about EJDeR and/or the Portal that you did not receive but that you wish you received before you started working? If so, what?  E) What did you think of the weekly clinical supervision? For example. frequency, duration, and clinical and technical content, respectively.  F) What did you think of the aids that were available via the web? For example. Portal Handbook, Padlet. |
| Practical layout, technology, and support | A) How did the communication with the research group work?  B) Did technical problems arise for your parents? If so, what? |
| Competence | A) How did you experience your competence in relation to the e-therapist role? |
| III. Closing questions | A) What have you learned from your work as an e-therapist?  B) Is there anything you want to add that we have not talked about? |
